# Information and Diagnosis Networks – tools to improve diagnosis and treatment for patients with rare genetic diseases

**DOI:** 10.1590/1678-4685-GMB-2018-0214

**Published:** 2019-06-10

**Authors:** Taiane Alves Vieira, Franciele Barbosa Trapp, Carolina Fischinger Moura de Souza, Lavínia Schuler Faccini, Laura Bannach Jardim, Ida Vanessa Doederlein Schwartz, Mariluce Riegel, Carmen Regla Vargas, Maira Graeff Burin, Sandra Leistner-Segal, Patrícia Ashton-Prolla, Roberto Giugliani

**Affiliations:** 1 Medical Genetics Service, Hospital de Clinicas de Porto Alegre, Porto Alegre, RS, Brazil; 2 Research and Postgraduate Group - Hospital de Clinicas de Porto Alegre, Porto Alegre, RS, Brazil; 3 Department of Genetics – Universidade Federal do Rio Grande do Sul, Porto Alegre, RS, Brazil; 4 Department of Internal Medicine - Universidade Federal do Rio Grande do Sul, Porto Alegre, RS, Brazil

**Keywords:** Information services, Medical Genetics, diagnostic networks, rare diseases, reference centers

## Abstract

Brazil is a country of continental dimensions and most genetic services are concentrated in the Southeast and South, including the Medical Genetics Service of the Hospital de Clínicas de Porto Alegre (MGS/HCPA). As many areas on the country do not have adequate medical genetics support, networks were designed to extend the service of the MGS/HCPA reference center. This paper presents the information and diagnosis networks that have their headquarters at MGS/HCPA: *SIAT* (National Information System on Teratogenic Agents), *SIEM* (Information Service on Inborn Errors of Metabolism), *Alô Genética* (Hello Genetics - Medical Genetics Information Service for Primary Health Care Professionals); *Rede MPS Brasil* (MPS-Mucopolysaccharidosis Brazil Network); *Rede EIM Brasil* (IEM-Inborn Errors of Metabolism Brazil Network), *Rede NPC Brasil* (Niemann-Pick C - NPC Brazil Network), *Rede DLD Brasil* (LSD-Lysosomal Storage Disorders Brazil Network), *Rede DXB* (MSUD-Maple Syrup Urine Disease Network), *RedeBRIM* (Brazilian Network of Reference and Information in Microdeletion Syndromes Project), *Rede Neurogenética* (Neurogenetics Network), and *Rede Brasileira de Câncer Hereditário* (Brazilian Hereditary Cancer Network). These tools are very useful to provide access to a qualified information and/or diagnostic service for specialized and non-specialized health services, bypassing difficulties that preclude patients to access reference centers.

## Introduction

Brazil is a country of continental dimensions and most services that deal with rare genetic diseases are concentrated in the Southeast and South regions, the most developed regions of the country. Such services are usually integrated to university hospitals and are responsible for providing medical care for thousands of patients and families every year (Horovitz *et al.*, 2013). This is the case of Medical Genetics Service of the Hospital de Clínicas de Porto Alegre (MGS/HCPA), located in South of Brazil and academically linked to the Federal University of Rio Grande do Sul (UFRGS).

MGS/HCPA is a service specialized in the diagnosis and treatment of rare genetic diseases, being a national (and international for some diseases) reference center for many conditions. MPS/HCPA is a WHO Collaborating Center and a hub for training and education in the field of medical genetics. Since 1990, the MGS/HCPA is developing remote support strategies addressed to the general public, health professionals, and medical personnel of the country, aiming to overcome barriers and provide access to information, diagnostic testing, and general guidance regarding rare genetic diseases. This paper presents a report on the activities of the information and diagnosis networks based at MGS/HCPA.

## SIAT

The SIAT, National Teratogen Information Service, was set up in 1990 at the MGS/HCPA as a project connected to UFRGS. Its primary objective is to provide pregnant women, obstetricians, and other health professionals from all over the country information on reproductive risks related to the exposure of pregnancies to pharmaceutical products and other chemical, physical, and biological agents. However, other objectives were incorporated including to generate knowledge about teratogenesis in humans with emphasis on exposures prevalent in Brazil and to transfer the knowledge on this area to both health professionals (with emphasis on the family health system and basic health care) and the community (by reinforcing the educational and prevention aspects).

Consultations are always done orally (usually over the phone) to a patient, but a written report is always sent to the assistant physician. The team that deals with the consultations is composed of doctors, biologists, and students from Medicine and Pharmacy. To date, SIAT has already answered approximately 10,000 consultations.

In addition to the consultations, the SIAT maintains proactive initiatives for primary prevention of congenital anomalies, such as systematic reviews of specialized literature on the field, lectures and presentations for health professionals, publications of books, book chapters, and research papers in specialized scientific journals, and epidemiological and laboratory research projects. Finally, SIAT maintains a webpage and a Facebook page where information is disseminated to the general population and to health professionals. Contact information is available in Table 1. Throughout these years, SIAT played an important role in the following items: (1) the identification of misoprostol, a medication widely used in Brazil in the 1990s, as a teratogenic agent in human species (Schuler *et al.*, 1992); (2) the identification of new cases of fetal thalidomide syndrome, with proposals for surveillance and prevention of exposures in Brazil including participation in the new national regulation of this product (Vianna *et al.*, 2017); (3) the follow-up of the rubella vaccination campaign in the state of Rio Grande do Sul (RS) and the demonstration of its safe use during pregnancy (Minussi *et al.*, 2008); (4) the follow-up of the H1N1 epidemic in RS (Silva *et al.*, 2014); (5) the active participation in the identification of the Zika virus as a teratogen in humans (Schuler-Faccini *et al.*, 2016).

**Table 1:** Information and Diagnosis Networks Summary.

Network	Contact information (Brazilian telephone numbers)	Focus
*SIAT* (National Information System on Teratogenic Agents)	(51) 3359-8008 www.gravidez-segura.org	Providing information on reproductive risks related to the exposure of pregnancies to pharmaceutical products and other chemical, physical, and biological agents. Target audience: pregnant women and health professionals
*SIEM* (Information Service on Inborn Errors of Metabolism)	0800 5102858 www.siem.ufrgs.br siem@ufrgs.br	Facilitating diagnosis and management of patients presenting any type of IEM. Target audience: health professionals
*Alô Genética* (Hello Genetics - Medical Genetics Information Service for Primary Health Care Professionals)	0800-642-6761 www.alogenetica.com alogenetica@ufrgs.br	Providing information about genetic diseases and guidelines regarding initial management and referral to specialized centers of patients who are suspected of presenting genetic diseases. Target audience: primary health care providers
*Rede MPS Brasil* (Mucopolysaccharidosis Brazil Network)	0800-510-2030 or 0800-645-2101 www.ufrgs.br/geneticahcpa/rede-mps/ mps@ufrgs.br	Improving the access to information, diagnosis, and treatment of MPS. Target audience: patients/families and health professionals
*Rede EIM Brasil* (Inborn Errors of Metabolism Brazil Network)	0800-510-2030 or 0800-645-2101 www.ufrgs.br/geneticahcpa/eim/ eim@ufrgs.br	Supporting the associated centers for diagnosis of IEM. Target audience: health professionals from participating centers
*Rede NPC Brasil* (Niemann-Pick C Brazil Network)	0800-510-2030 or 0800-645-2101 npc@ufrgs.br	Providing information and access to diagnostic tests of NPC. Target audience: health professionals
*Rede DLD Brasil* (Lysosomal Storage Disorders Brazil Network)	0800-510-2030 or 0800-645-2101 www.ufrgs.br/geneticahcpa/dld/ dld@ufrgs.br	Supporting the diagnosis of LSD patients, enabling them to access the management measures available. Target audience: health professionals
*Rede DXB* (Maple Syrup Urine Disease Network)	www.redexaropedobordo.com.br	Supporting research, diagnosis, and management of MSDU. Target audience: patients/families and health professionals
*RedeBRIM* (Brazilian Network of Reference and Information in Microdeletion Syndromes Project)	(51) 3359-8011 http://www.ufrgs.br/geneticahcpa/	Joining efforts for the diagnosis and research in chromosomal microdeletions associated with malformation syndromes and intellectual disability. Target audience: health professionals from participating centers
*Rede Neurogenética* (Neurogenetics Network)	www.redeneurogenetica.ufrgs.br	Getting epidemiological information about mendelian neurodegenerative diseases, mainly spinocerebellar ataxias. Target audience: researchers from Brazil and Peru
*Rede Brasileira de Câncer Hereditário* (Brazilian Hereditary Cancer Network)	(51) 3359-8011	Advancing knowledge about hereditary cancer across health care disciplines and facilitating access to patient care in the field. Target audience: health professionals

SIAT is a consolidated initiative of the MGS/HCPA with important scientific and social contributions, and has been a model for similar initiatives aiming prevention of birth defects and provision of information about teratogens in other parts of Brazil and in neighboring countries.

## SIEM

The Information Service on Inborn Errors of Metabolism (SIEM) has been operating at MGS/HCPA since October 2001. SIEM is a pioneer effort in the area of Inborn Errors of Metabolism (IEM), and we are not aware of any similar information service in Brazil or in Latin America (Brustolin *et al.*, 2006). The service operates 24/7, with personal assistance from Monday to Friday 9:00-12:00 and 14:00-17:00 (in other days/times the request is recorded and answered as soon as possible). Contact information is available in Table 1.

All incoming cases are studied and discussed by SIEM’s team. Once contacted by a health professional, a form is completed with relevant clinical information that is registered in a database and analyzed by a specialist who will suggest diagnostic hypotheses, further laboratory and/or imaging procedures, and, if necessary, emergency management measures. Feedback with guidance could be given promptly (in case of urgency) or within a maximum of 48 hours after the original contact.

The main objective of this toll-free service is to help physicians and health professionals involved in the diagnosis and management of patients presenting any type of IEM. Phone consultations are attended by professionals with training in IEM, with the support of a multidisciplinary team that includes geneticists, pediatricians, biochemists, molecular biologists, and dieticians. In order to record data on the investigation and diagnostic conclusion of each case, follow-up assessments are conducted up to three months after the original phone or email contact. All cases are recorded on a specific database. Cases are considered “finalized” after diagnosis has been confirmed, and the etiology classified as metabolic or non-metabolic. Despite all efforts, some cases may remain undiagnosed or inconclusive.

**Figure 1:**
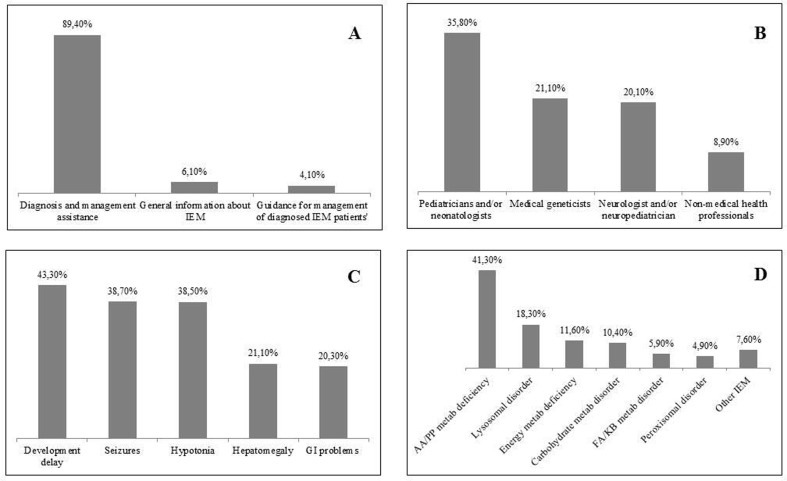
Data from 3277 cases recorded by SIEM from 2001 to 2017. (A) Type of request, (B) Professionals who contact the service, (C) Most frequent signs and symptoms, (D) Conclusive diagnosis of 2658 cases. Data is shown in percentage. EIM - Inborn Error of Metabolism; GI – Gastrointestinal; AA – Amino acid; PP – Peptide; FA – Fatty acid; KB – Ketone bodies.

From 2001 to 2017, 3277 cases were recorded. Figure 1A shows the type of requests that professionals presented to SIEM. Figure 1B shows the professionals who contact the Service. The purpose of the contacts were mostly related to symptomatic patients in whom an IEM was suspected (86.5%); the most frequent symptoms registered are shown in Figure 1C. The onset of symptoms occurred usually before one year of age (68.4%). A conclusion was reached in 2658 cases (81.1%), being 329 (12.4%) confirmed as an IEM, distributed as shown in Figure 1D.

This distribution exemplifies the heterogeneity of the IEM, individually very rare but not that rare when considered as a group. Diagnosis is challenging due to non-specific symptoms and to the need of specialized tests; however, it needs to be established as early as possible to allow an adequate and accurate management of the patient, to try to decrease the burden of the disease (Giugliani, 2010). The SIEM has been an important center for specialized information and guidance about inherited metabolic diseases.

## Hello Genetics (Alô Genética)

“Hello Genetics” is an information service designed to support Primary Health Care (PHC) professionals seeking information about genetic diseases. This service emerged after a pilot project for testing the integration of medical genetics into PHC, which demonstrated the need for continued support to these professionals (Vieira *et al.*, 2013). This service aims to provide to PHC providers information on genetic diseases and guidelines regarding initial management and referral to specialized centers of patients who are suspected of presenting genetic diseases.

Brazilian PHC providers can contact the service by calling the nationwide toll free number, sending an e-mail, or accessing the website. Contact information is available in Table 1. The first contact is with a trained staff member who fills out a standard form with the information provided by the PHC professional, including the main question that generated the service request (information on genetic diseases or guidelines on initial management). Afterwards, the file is analyzed by a trained nurse who, together with a clinical geneticist, prepares the report that is sent to the requesting PHC provider in no more than 7 days.

In partnership with Telessaúde (a program that provides remote support to the Brazilian Public Health System - SUS), “Hello Genetics” was officially included in the evaluation process of consultation requests in medical genetics in specialized centers that provide services for the SUS.

Consistent with the proposal by the National Policy for Comprehensive Attention for Persons with Rare Diseases (Brasil, 2014), which establishes that PHC should promptly refer the person with a suspected rare disease for diagnostic confirmation, “Hello Genetics” comes as a promising service to fill a gap between PHC and specialized medical genetics care. Through this tool, PHC providers can obtain information about genetic conditions in order to provide an initial guidance to patients and families with or at risk of presenting genetic diseases, and to make decisions about referral to reference services, contributing to facilitate the access of SUS users to specialized care.

## MPS Brazil Network (Rede MPS Brasil)

The mucopolysaccharidoses (MPS) are a group of lysosomal storage disorders (LD) characterized by intralysosomal storage of glycosaminoglycans (Neufeld and Muenzer, 2001). In 2004, the MPS Brazil Network was set up aiming to improve the access of families and health professionals to information, diagnosis, and treatment of MPS.

The MPS Brazil Network is a partnership of medical services in Brazil that deal with MPS patients. The network’s webpage provides a wide range of information and a tool for the request of diagnostic tests, which are performed in the network’s laboratories. Contact information is available in Table 1. The MPS Brazil Network publishes a quarterly newsletter named “*Caiu na Rede*”, with testimonials from health care professionals, feedback about available therapies, news on clinical trials, meeting reports, and anything that may interest the MPS community.

Together with family associations (AGMPS) and non-governmental organizations (Genetics for All Institute, IGPT), regular meetings are organized by the MPS Brazil Network to keep families updated with the most recent advances in the field.

**Figure 2:**
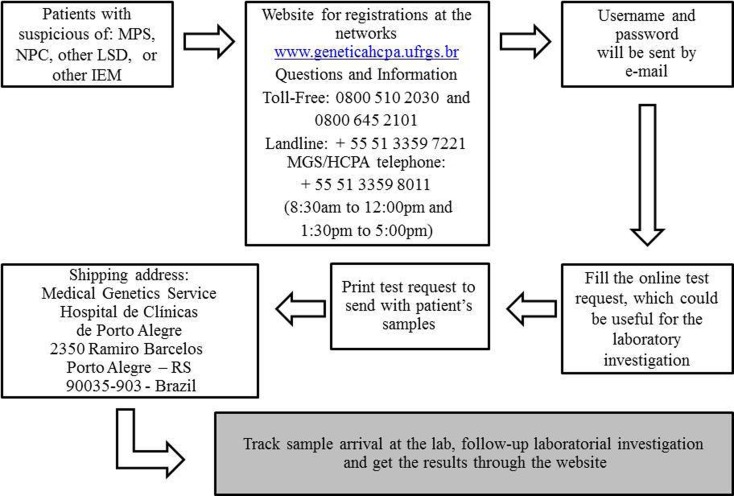
Diagnostic Networks flowchart showing the operation from the clinical suspicion to the analysis and follow-up of the samples (common to MPS, IEM, NPC, LSD, and MSUD Networks).

This initiative is supported by public and private funds that enable the provision of the services free of charge, making information and diagnostic tests available even for families that usually do not have access to sophisticated healthcare facilities (Giugliani, 2012). Figure 2 provides a flowchart illustrating the operation of the information and diagnostic networks, common to the MPS Brazil Network and to other networks with the same administrative management (IEM Brazil Network, NPC Brazil Network, LSD Brazil Network, and MSUD Network).

Since its creation in 2004, the MPS Brazil Network has been able to support physicians of all Brazilian regions and reach 16 countries, facilitating the access to diagnosis of MPS. By 2017, approximately 1400 MPS patients were diagnosed (MPS I: 260; MPS II: 401; MPS III-A: 62; MPS III-B:102; MPS III-C: 67; MPS III-D: 0; MPS IV-A: 190; MPS IV-B:13; MPS VI: 278; MPS VII: 22).

We hope that this initiative will continue to identify patients with MPS in Brazil and abroad, contributing to their management, and increasing their quality of life and life expectancy.

## IEM Brazil Network (Rede EIM Brasil)

Inborn errors of metabolism (IEM) are individually rare, but collectively numerous. From a pathophysiological perspective, metabolic disorders can affect the synthesis, degradation, processing, and transportation of molecules in the organism (Fernandes *et al.*, 2006).

Because of the difficulties related to the sophisticated diagnostic methods, the identification of IEM is a challenge for clinicians attempting to diagnose and manage these conditions in Brazil. The IEM Brazil Network was set up in 2006 with the aim of supporting the associated centers for diagnosis of IEM. Contact information is available on Table 1.

As the cost of the diagnostic tests provided by the network is supported by research projects and the laboratory has a limited operational capacity, each request is reviewed by the technical staff before the samples are authorized for shipping to the laboratory (not applicable in urgent situations). The diagnostic service of the IEM Brazil Network is provided only to patients who are being evaluated by a public health system (SUS) associated service.

Having diagnosed over 1800 patients with IEM since its creation in 2006, the IEM Brazil Network is also helping to map the IEM incidence in each region of the country, contributing with the identification of clusters, which could be evaluated through particular projects and help health authorities to plan actions for specific areas.

## NPC Brazil Network (Rede NPC Brasil)

Niemann-Pick disease type C (NP-C) is a rare neurodegenerative lysosomal storage disorder characterized by intracellular lipid trafficking abnormalities (Vanier, 1997). In order to improve the access to its diagnosis, the NP-C Brazil Network was set up in 2009 aiming to provide physicians from several specialties with screening and diagnostic tests to identify NP-C patients in Brazil and other countries.

The NPC Brazil Network can be accessed by any service that evaluates patients who could present NP-C. Contact information is available on Table 1. The initiative also develops research projects about NP-C and supports educational actions related to this disease.

Since 2009, more than 2000 samples from at-risk patients were evaluated. The protocol includes oxysterols and chitotriosidase assays, Filipin test, measurement of enzyme activities that could provide differential diagnosis (b-glucosidase, sphyngomyelinase, acid lipase), and a final confirmation of the diagnosis with sequencing of the NPC1 and NPC2 genes. The investigations allowed the diagnosis of more than 100 NP-C patients from 2009 to 2017, allowing specific and general management measures for the affected subjects and families.

## LSD Brazil Network (Rede DLD Brasil)

Lysosomal storage diseases (LSDs) are conditions caused by a defect in lysosomal function (Wilcox, 2004). Due to the significant importance of this group of diseases, a large part of which are treatable, the LSD Brazil Network was set up in 2012 following the successful examples of MPS, IEM, and NPC networks.

The aim of this network is to support the diagnosis of LSD patients, enabling them to access the management measures available. Any medical service of the country can access the network through phone, e-mail, or webpage, according to contact information available on Table 1. The initiative also supports educational actions related to this disease and works on research projects related to LSD.

It is important to highlight that the establishment of the LSD Brazil Network was probably a leading factor for the significant increase in LSD cases diagnosed at MGS/HCPA in the last years, allowing the estimation of the minimal incidence of LSDs in Brazil, which could be useful to health authorities, as there are specific therapies available for many of these conditions (Giugliani *et al.*, 2017).

## MSUD Network (Rede DXB)

The Maple Syrup Urine Disease Network (MSUD Network) was created in 2014. The existence of a specific network for a specific rare disease (MSUD, for which the Portuguese acronym is DXB) is justified by the fact that early diagnosis and treatment of this disease prevent intellectual disability and death in affected patients (usually children). Since MSUD is not part of the official Brazilian Neonatal Screening Program, and since its manifestations are generally confused with those of more frequent diseases, Brazilian patients are often diagnosed late and therefore have sequelae.

Thus, the main goal of the MSUD Network is structuring an organized national network of referral and counter-referral for MSUD research, diagnosis, and management considering the perspective of the SUS.

Since its inception, the MSUD Network has registered 133 patients from all Brazilian regions, 13 of them submitted to liver transplantation. The mean age of the first sample analyzed by the laboratory was nine months, confirming the diagnostic lateness of this disease is in our country. In the year 2017, an average of 26 quantitative amino acid analyses in blood were performed per month for diagnostic and follow-up purposes.

The MSUD Network also has a character (a girl called Lina) created exclusively to help educate the population about the MSUD. In addition, the Network has its own website (available on Table 1) and a Facebook page, where information about the disease can be found including patient care centers, research projects, and centers that participate in the Network, being an important channel of communication and support for health professionals, relatives, and patients.

In the last four years, six articles were published in international journals, describing the panorama of the disease in the country (Herber *et al.*, 2015) and providing innovative scientific information on pathophysiological mechanisms (Scaini *et al.*, 2017, 2018) and new treatment strategies, such as “domino” liver transplantation (Feier *et al.*, 2014, 2016; Roda *et al.*, 2018).

## RedeBRIM (Microdeletion Network – Rede de Microdeleções)

The estimated overall frequency of microdeletion syndromes (MS) is over 1:1000 liveborns (Schinzel *et al.*, 2003; Vissers and Stankiewicz, 2012). Although the clinical picture of many MS is well defined, the diagnostic facilities are available only in few centers that operate in the SUS in Brazil; systematic studies are limited to some specific common microdeletions, and the actual overall frequency of these syndromes in Brazil has not been reported.

With the main goal of joining efforts for the diagnosis and research in chromosomal microdeletions associated with malformation syndromes and intellectual disability, we proposed in 2011 a retrospective Brazilian collaborative multicenter study. RedeBRIM, the Brazilian Network of Reference and Information in Microdeletion Syndromes Project, is formed by a reference center (MGS/HCPA) and 15 participating centers that operate in the SUS. Contact information is available in Table 1.

From 2011-2017 a total of 1828 samples with an indication of chromosomal rearrangement were referred for molecular cytogenetic investigation. Of these, 1058 cases had a specific clinical indication for the investigation of a chromosome microdeletion (Table 2).

**Table 2:** Demographic, clinical, and FISH findings obtained from subjects referred to cytogenetic investigation of specific (micro) deletion syndromes (2011-2017).

Syndrome (OMIM)	Age range	M	F	Most frequent clinical features reported in the hospital records	Locus	n (%)	deleted/n (%)
AS	1y-28y	66	88	DD/ID, ataxic gait, inappropriately happy disposition, hypotonia, microcephaly, profound speech impairment, seizures	15q11.2	154 (14.55)	8/154 (5.19)
CdCS (123450)	4m-35y	16	18	High-pitched monotonous cry microcephaly, hypertelorismepicanthic folds, round face, severe DD, and learning disabilities	5p15.2	34 (3.21)	27/34 (19.41)
LGS (190351)	4m-20y	7	3	Long flat philtrum ID, exostoses, cone-shaped epiphyses	8q24.12	10 (0.94)	8/10 (80)
22qDS (188400) (192430)	NB- 40y	176	168	Congenital heart defects/ Conotruncal and aortic arch, facial dysmorphic features, DD	22q11.2	344 (32.51)	120/344 (34.88)
MDS (247200)	2m-30y	17	6	Microcephaly, growth retardation, DD/ID with seizures and EEG abnormalities	17p13.3	23 (2.17)	3/23 (13.04)
PWS (176270)	3m-43y	118	124	ID, postnatal hipotonia, obesity due to food seeking, hypogonadotrophic hypogonadism	15q11.2	242 (22.87)	32/242 (13.22)
RTS (180849)	1y-49y	11	7	ID, broad thumbs and toes facial dysmorphism	16p13.3	18 (1.70)	2/18 (11.11)
SoS (606681)	NB-17y	9	10	DD, increased birth length and weight, excessive growth in childhood	5q35	19 (1.79)	2/19 (10.52)
WBS (194050)	1m-39y	88	74	DD/ID, overfriendliness, congenital heart disease, specially SVAS, facial characteristic including bulbous nasal tip, wide mouth, full lips, full cheeks and small widely spaced teeth	7q11.23	162 (15.31)	104/162 (64.19)
WHS (194190)	NB-39y	11	27	A “Greek-helmet” profile, low birth-weight and postnatal failure to thrive, microcephaly, DD	4p16.3	38 (3.59)	36/38 (94.73)
SMS (182290)	3y-19y	7	7	DD, learning disability, behavioral disturbance, facial characteristics	17p11.2	14 (1.32)	3/14 (21.42)
Total (%)	-	526(49.71)	532(50.28)	DD/ID	-	1058 (100)	-

AS: Angelman syndrome; CdCS: Cri-du-Chat syndrome; LGS: Langer Giedion syndrome; 22qDS, 22q11.2 deletion syndrome; MDS: Miller-Diecker syndrome; PWS: Prader-Willi syndrome; RTS: Rubinstein-Taybi syndrome; SoS: Sotos syndrome; WBS: Williams-Beuren syndrome; WHS: Wolf-Hirschhorn syndrome; SMS: Smith-Magenis syndrome; OMIM: Online Mendelian Inheritance in man; NB: newborn; m: months; y: years; DD: developmental delay; EEG: electroencephalogram; ID: intellectual disability; SVAS: supravalvular aortic stenosis; M: male; F: female; n: total.

This initiative contributed to the establishment of an organized network for cytogenetic research and diagnostics involving public health services in Brazil. The diagnostic and research activity developed by RedeBRIM also attracted the attention of many young fellows looking for training opportunities in the field. This led to the creation of a comprehensive capacitation program in cytogenetics, developed under the umbrella of the UFRGS and SGM/HCPA. This program provides short-term (4-6 months) and long term (12-24 months) training in cytogenetics for health professionals, academic postgraduate capacitation and also scientific initiation opportunities.

The development of genomic technologies has changed the cytogenetics diagnostic practice along the last decade. Hence, it is mandatory that cytogenomic approaches are incorporated also in the public health system in Brazil. While this is not a reality yet in the majority of genetic centers, alternative proposals such as the RedeBRIM project have been providing laboratory diagnosis, supporting capacitation programs and continuing education of cytogeneticists, contributing to a better knowledge of chromosomal rearrangements associated with genomic disorders.

## Neurogenetics Network (Rede Neurogenética)

The Neurogenetics Network is a collaborative group of Brazilian and Peruvian scientists whose research emphasis is on Mendelian neurodegenerative diseases (website available on Table 1). The main focus of this network were the spinocerebellar ataxias (SCAs) SCA1, SCA2, SCA3 (Machado Joseph disease), SCA6, SCA7, SCA10, SCA12, SCA17, Dentatorubral-pallidoluysian atrophy, Huntington disease (HD), Myotonic Dystrophy type 1, Friedreich ataxia, Neuronal Ceroid Lipofuscinosis type 3, and familial transthyretin amyloidosis. As rare and chronically debilitating diseases, these conditions are of “such low prevalence that special combined efforts are needed to address them” (European Commission, 2017). This network started in 2008 with the original aims to get epidemiological information about these disorders in Brazil at first, and after that, to address specific research questions related to the most relevant epidemiological findings. Indeed, more than 1300 subjects with a clinical picture suggestive of these conditions were recruited at university hospitals from several Brazilian cities and from Lima, Peru.

A portrait of SCA patients (363 families) from Brazil was outlined (Castilhos *et al.*, 2014) when 216 families were identified with SCA3 (59.5%), 28 with SCA2 (7.7%), 20 with SCA7 (5.5%), 15 with SCA1 (4.1%), 12 with SCA10 (3.3%), 5 with SCA6 (1.4%), while 66 families remained undiagnosed (18.2%). Several other research lines followed this initial report. Some of them aimed to relate novel clinical findings - for instance, the relationship between spastic paraplegia and SCA1 (Pedroso *et al.*, 2015). A longitudinal study was performed using a large number of SCA2 symptomatic carriers; this study had sufficient power to detect changes in the natural history as never seen before (Pereira *et al.*, 2015; Monte *et al.*, 2017a,b,c). Of note, identification of several SCA10 families – a disorder limited to the Americas – brought to light ancestral origins of this condition (Gheno *et al.*, 2017; Bampi *et al.*, 2017).

The proportion of genetic diagnoses obtained among 104 Brazilian families with HD phenotype was described in another pioneer, large cross-sectional study performed by Rede Neurogenética (Castilhos *et al.*, 2014). There were 93 HD, 4 HDL2, and 1 SCA2 families, while no cases of HDL1, SCA17, DRPLA, neuroferritinopathy, benign hereditary chorea, or CHAC were found.

Several projects are in progress (Table 3), which we believe will open new perspectives related to epidemiology and clinical relevance, and improve knowledge about neurogenetic disorders in Brazil as well as in Latin America in the years to come.

**Table 3:** Research in progress related to Rede Neurogenetica (status in December, 2017).

Disease	Scientific question	Participant sites	Perspective
Huntington disease	Mutation transmission and Minimal prevalence	Rio Grande do Sul, São Paulo and Rio de Janeiro states, Brazil	Submitted
	Carnitine and Branched Chain Amino Acids as state biomarkers	Rio Grande do Sul, São Paulo and Rio de Janeiro states, Brazil	Submitted
Spinocerebellar ataxias in general	Proportion of diagnoses in Peruvian populations	Rio Grande do Sul and Peru	Finished Paper will be submitted
Spinocerebellar ataxia type 3/ Machado Joseph disease	Ancestral origins	Rio Grande do Sul, Santa Catarina, São Paulo, Rio de Janeiro, Paraíba, Rio Grande do Norte, and Pará states, Brazil	Finished Paper will be submitted
Spinocerebellar ataxia type 7	Ophthalmologic and neurologic findings as state biomarkers in symptomatic and pre-symptomatic stages.	Rio Grande do Sul and Rio de Janeiro states, Brazil	Submitted
	Ancestral origins	Rio Grande do Sul, São Paulo and Rio de Janeiro states, Brazil	Not recruiting yet.
Spinocerebellar ataxia type 2	Progression rate of cognitive losses	Rio Grande do Sul, São Paulo and Rio de Janeiro states, Brazil	Finished Paper will be submitted

## Brazilian Network of Hereditary Cancer (Rede Brasileira de Câncer Hereditário)

The development of Clinical Cancer Genetics in Brazil over the past twenty years has advanced and implemented comprehensive strategies to provide standards for counseling, surveillance, genetic testing, and patient care, using as model high-structured programs in North America and Europe (Palermo *et al.*, 2007). Despite these advances, the current coverage of comprehensive oncogenetic services that include genetic testing is restricted to less than 30% of the Brazilian population that has private insurance; even for those, shortage of health care providers trained in the field and lack of awareness is a significant limitation. For patients of the SUS, genetic testing is not yet available, although some universities and academic centers provide clinical cancer risk assessment and genetic counseling.

The Brazilian Hereditary Cancer Network (ReBraCH) was initially supported by public research funding (National Council for Scientific and Technical Development, CNPq). This network is a consortium of 12 institutions dedicated to the advance of knowledge about hereditary cancer across health care disciplines and facilitate access to patient care in the field. The network organizes specialized training in hereditary cancer and has developed educational materials as well as a wealth of information on the molecular epidemiology of hereditary cancer syndromes in Brazil (Brasil, 2009).

Participating centers are mainly localized in public health care institutions, and currently, perform approximately 8000 outpatient consultations per year. However, this coverage is still limited because these centers are located in urban areas far from large part of the rural population and because their efficacy is constrained by shortage of properly trained medical and non-medical health care professionals. Moreover, as SUS does not contemplate financial support for genetic testing for hereditary cancer syndromes, access to testing is limited. A significant advance, however, took place in 2012, when the coverage of genetic testing by private health care plans became mandatory in Brazil.

Major challenges lay ahead for the Brazilian Familial Cancer Network in enabling access for clinical cancer genetics services, especially for patients from the public health care system in Brazil (Ashton-Prolla and Seuanez, 2016). These include disseminating the importance of genetic testing for all, disseminating information to remote areas of the country, and creating career opportunities for health care professionals by providing comprehensive training programs in cancer genetics. Development and implementation of novel diagnostic technologies, multidisciplinary approaches to patient care, and collaborative research projects must be stimulated, aiming at a more comprehensive understanding of hereditary cancer in Brazil.

## Conclusion

The operation of the information and diagnostic networks have been very useful in providing support for medical genetics, as well as improving the access to diagnosis to patients countrywide, especially considering the geographic characteristics of our country and the concentration of genetics services and laboratories in southern and southeastern regions. The possibility of requesting specific laboratory tests directed for the diagnosis of genetics conditions and the contact with specialized staff can lead to quicker diagnoses, providing a shortcut to the “diagnostic odyssey” faced by families with rare diseases. Furthermore, the networks can also refer patients to each other to provide a better approach for selected cases.

In addition, the information and diagnostic networks enhance partnership and collaboration among health institutions, promote multi-disciplinary research, facilitate the generation of clinical and epidemiological data on rare genetic diseases, and help better understand the care needed by patients and families affected by these conditions.

As exposed in this paper, a large number of diagnoses was confirmed by the networks, as well as a large number of enquiries was addressed. The MGS/HCPA will continue contributing to the improvement of the access to information and diagnosis on rare genetic diseases, enabling patients and families to benefit from the management measures available for these conditions. These networks can provide an interesting model for implementing comprehensive support strategies in other countries.
